# Association between Polymorphisms of X-Ray Repair Cross Complementing Group 1 Gene and Pancreatic Cancer Risk: a Systematic Review with Meta-Analysis

**DOI:** 10.1007/s12253-017-0364-6

**Published:** 2017-12-28

**Authors:** Jun Chen, Hong Wang, Zhiming Li

**Affiliations:** 1 Department of General Surgery, Affiliated Hospital of Traditional Chinese Medicine, Xinjiang Medical University, No. 116 the Yellow River Road, Urumqi, 830000 China; 2 Department of Surgery, Shihezi Hospital of traditional Chinese Medicine, Shihezi, China

**Keywords:** XRCC1, Polymorphisms, Pancreatic cancer, Meta-analysis

## Abstract

Emerging evidences have shown that common genetic polymorphisms in X-ray repair cross complementing group 1 (XRCC1) gene may be associated with the development of pancreatic cancer, but individually published studies and previous meta-analyses revealed inconclusive results. The aim of our study was to investigate the association between polymorphisms in XRCC1 gene and pancreatic cancer risk. We conducted a search of PubMed, Embase, the Cochrane Library and Web of Science databases. Odds ratios (ORs) with 95% confidence intervals (CIs) were determined as measures of the strength of association between polymorphisms of XRCC1 and pancreatic cancer risk. Sensitivity analysis and publication bias were evaluated. All analyses were undertaken using the STATA 13.0. A total of 10 studies were included in this systematic review. Five common functional single-nucleotide polymorphisms (SNPs) in XRCC1 gene were found, including Arg399Gln G > A (rs25487), Arg194Trp C > T (rs1799782), Arg280His G > A (rs25489), c.1517G > C, c.1471G > A. Results from our stratified analysis based on Hardy-Weinberg equilibrium (HWE) showed that there was a robust significant association between Arg280His polymorphism and pancreatic cancer risk (allelic model, OR 0.743, 95% CI 0.576–0.958, *P* = 0.022; heterozygous model, OR 0.701, 95% CI 0.525–0.936, *P* = 0.016; dominant model, OR 0.710, 95% CI 0.537–0.939, *P* = 0.016). We also found a statistically significant association between c.1517G > C polymorphism and pancreatic cancer risk (Allelic model, OR 1.252, 95% CI 1.064–1.473, *P* = 0.007). No significant results were obtained for Arg399Gln, Arg194Trp and c.1471G > A polymorphisms. The present meta-analysis suggested that Arg280His and c.1517G > C polymorphisms in XRCC1 gene were associated with pancreatic cancer risk.

**Electronic supplementary material:**

The online version of this article (10.1007/s12253-017-0364-6) contains supplementary material, which is available to authorized users.

## Introduction

Pancreatic cancer, although infrequent, has an exceptionally high mortality rate. The development of pancreatic cancer is a complex and multifactorial process [1], and has many associated risk factors, such as include high-fat diet, smoking, chronic pancreatitis, primary sclerosing cholangitis, hereditary pancreatitis, family history of pancreatic cancer and diabetes mellitus [2]. It is well known that the genetic factors play key roles in the development of pancreatic cancer [1].

DNA repair systems are essential for maintaining the integrity of the genome and play key roles in protecting against mutations. The single nucleotide polymorphisms (SNPs) in DNA repair genes can cause the differences in the DNA repair capacity, which may contribute to the development of cancer because reduced DNA repair capacity may lead to genetic instability and carcinogenesis [3, 4]. It has been well accepted that the SNPs in the base excision repair (BER) gene can change the individual repair capacity in response to DNA damage [5]. X-ray repair cross-complementation group 1 (XRCC1) gene that is located on chromosome no. 19q13.2–13.3 plays a critical role in the BER of DNA damage [6]. There are more than 300 validated SNPs in the XRCC1 gene reported in the dbSNP database (http://www.ncbi.nlm.nih.gov/SNP/). Several SNPs in XRCC1 are associated with several types of cancer cancers risk, such as breast cancer [7], hepatocellular carcinoma [8] and lung cancer [9]. Recently, several studies have investigated the association between SNPs in XRCC1 gene and pancreatic cancer risk. However, results remain controversial in part because of the differences from the sample sizes. In this study, five common functional single-nucleotide polymorphisms (SNPs) in XRCC1 gene were found, including Arg399Gln G > A (rs25487), Arg194Trp C > T (rs1799782), Arg280His G > A (rs25489), c.1517G > C, c.1471G > A. For better understanding of the effects of the five SNPs in XRCC1 gene on pancreatic cancer risk, we conducted a systematic review with meta-analysis of previous published studies.

## Materials and Methods

### Identification of Eligible Studies

This meta-analysis was conducted according to PRISMA statement [10] (S1 PRISMA Checklist) and Cochrane Collaboration guidelines (http://handbook.cochrane.org/). We performed a systematic search using PubMed, Embase, the Cochrane Library and Web of Science with the last search updated on February 12, 2017. The search strategies were based on combinations of the following terms: “XRCC1 or rs25487 or Arg399Gln or rs1799782 or Arg194Trp or rs25489 or Arg280His or c.1517G>C or c.1471G>A” AND “pancreatic cancer or pancreatic carcinoma”. In addition, all references of included studies and previously published reviews were manually screened and reviewed for relevant studies.

### Eligibility Criteria

Studies were included in this meta-analysis if they satisfied the following criteria: (1) It was a case-control study; (2) The study assessed the association between XRCC1 gene (Arg399Gln, Arg194Trp, Arg280His, c.1517G > C, or c.1471G > A) polymorphisms and pancreatic cancer risk; (3) The study provided complete genotypes distribution data for estimating the odds ratios (ORs) and 95% confidence intervals (95% CIs); (4) Genotyping method in each study was universally acknowledged. The exclusion criteria of studies were as follows: (1) duplication of the previous publications, (2) abstract, comment, case reports, letters, and review, and (3) no sufficient data were provided. Two reviewers independently identified eligible studies according to the selection criteria. Disagreements were resolved by reaching a consensus among all authors.

### Data Extraction

Data were extracted by two reviewers independently, and then another reviewer verified them and any discrepancies were resolved by consensus. The following data were extracted from each study: the first author’s name, publication year, country of origin, ethnicity, genotyping methods, numbers of cases and controls, genotype frequency in cases and controls, respectively.

### Statistical Analysis

Hardy-Weinberg equilibrium (HWE) was evaluated by Pearson’s goodness-of-fit χ^2^ test for each study in the controls, and a *P* value of less than 0.05 was considered as deviation from HWE [11]. The strength of the association between the XRCC1 gene polymorphisms and pancreatic cancer risk was assessed by calculating crude odds ratios (ORs) along with their 95% confidence intervals (CIs). The pooled ORs (Arg399Gln, Arg280His and c.1471G > A polymorphisms) were calculated for the allelic model (A versus G), heterozygous model (GA versus GG), homozygous model (AA versus GG), dominant model (AA + GA versus GG) and recessive model (AA versus GA + GG). The pooled ORs (Arg194Trp polymorphism) were calculated for the allelic model (T versus C), heterozygous model (CT versus CC), homozygous model (TT versus CC), dominant model (TT + CT versus CC) and recessive model (TT versus CT + CC). Similarly, the pooled ORs (c.1517G > C polymorphism) were calculated for the allelic model (C versus G), heterozygous model (GC versus GG), homozygous model (CC versus GG), dominant model (CC + GC versus GG) and recessive model (CC versus GC + GG). Heterogeneity was determined using the Cochrane’s Q test and I^2^ statistics [12]. When the Q-test showed a *P* < 0.05 or I^2^ test exhibited >50%, the random-effect model was used for the meta-analysis [13]; otherwise, the fixed-effect model was chosen [14]. Sensitivity analyses were also carried out by sequential omission of each study one at a time to ensure the stability of our results. Publication bias was assessed using Begg’s funnel plot [15] and Egger’s linear regression method [16]. A *P* value of less than 0.05 was considered representative of statistically significant publication bias. All statistical analyses were performed by using STATA version 13.0.

## Results

### Study Characteristics

**Fig. 1:**
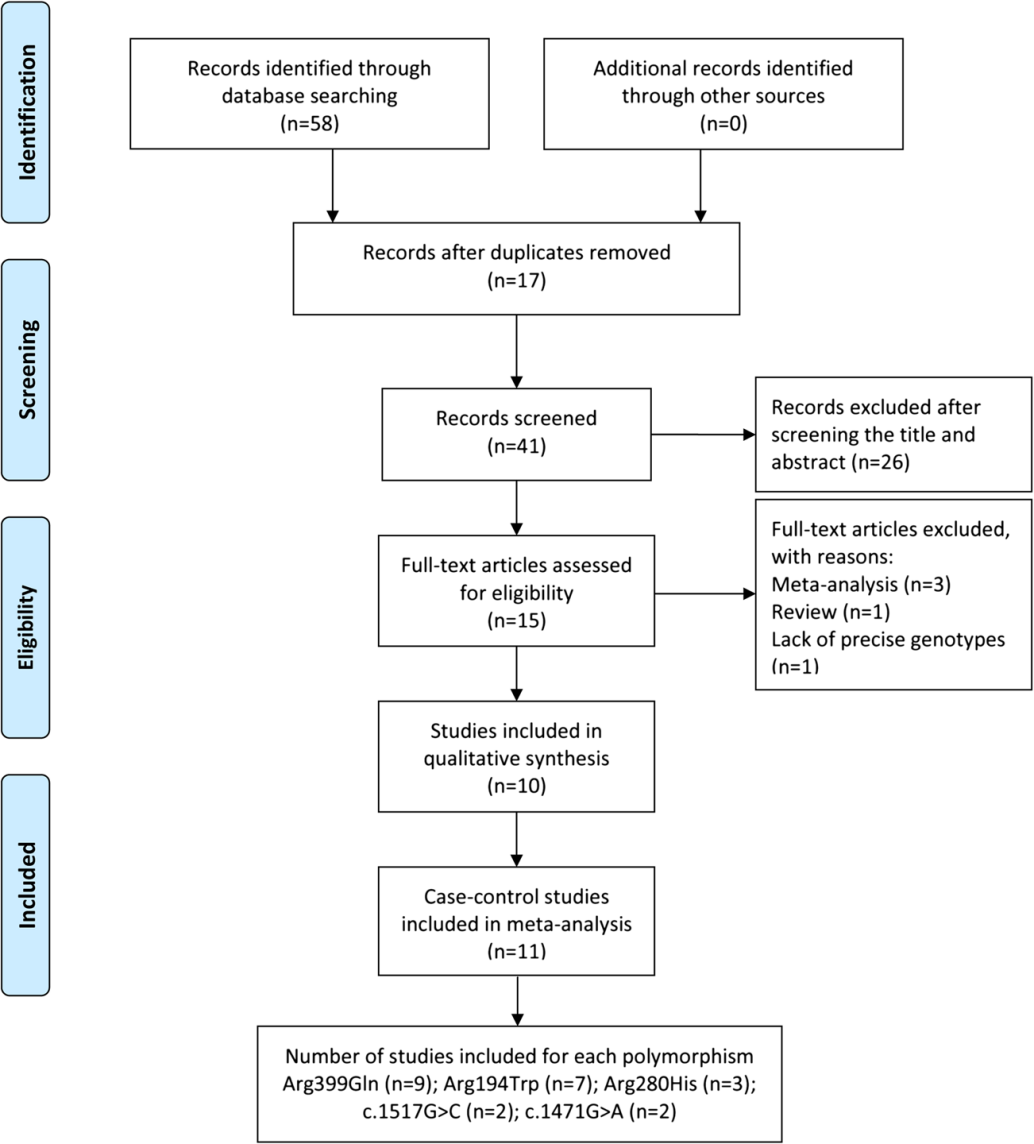
Flowchart of study selection

We give the detailed search strategies and results in the supplemental material (see S1 Table in the supplemental material). A total of 58 records were initially identified from PubMed, Embase, the Cochrane Library and Web of Science. Based on the eligibility criteria, a total of 10 studies (8 studies in English and 2 in Chinese) were included in this systematic review. In the end, 9 case-control studies for Arg399Gln polymorphism, 7 case-control studies for Arg194Trp, 3 case-control studies for Arg280His, 2 case-control studies for c.1517G > C, and 2 case-control studies for c.1471G > A were selected for this meta-analysis. The flow diagram summarizing the literature review process and final participation is shown in Fig. 1. The main characteristics of the selected studies are presented in Table 1.

**Table 1:** Characteristics of studies included in the meta-analysis

Study	Year	Country	Ethnicity	Genotyping method	Sample size		SNP
					Cases	Controls	
Wang LJ	2016	China	Asians	PCR-RFLP	152	264	Arg399Gln; Arg280His; Arg194Trp
Hou BH	2016	China	Asians	PCR-RFLP	298	298	Arg399Gln; Arg280His; Arg194Trp; c.1517G > C; c.1471G > A
Zhao ZM	2014	China	Asians	PCR-RFLP	390	392	c.1517G > C
Yan D	2013	China	Asians	SNaPshot	210	213	Arg399Gln; Arg194Trp
Chen H	2013	China	Asians	CRS-PCR	328	350	c.1471G > A
Nakao M	2013	Japan	Asians	TaqMan	185	1465	Arg399Gln; Arg194Trp
Mcwilliams RR	2008	USA	Caucasians	PCR	481	625	Arg399Gln; Arg280His; Arg194Trp
Wang L	2006	China	Asians	PCR	101	337	Arg399Gln; Arg194Trp
Jiao L	2006	USA	Caucasians	PCR	384	357	Arg399Gln; Arg194Trp
Duell EJ	2002	USA	Caucasians	PCR-RFLP	261	860	Arg399Gln
Duell EJ	2002	USA	Asians	PCR-RFLP	17	53	Arg399Gln

*PCR*, polymerase chain reaction; *RFLP*, restriction fragment length polymorphism; *CRS-PCR*, created restriction site-PCR; *SNP*, single nucleotide polymorphism

### Meta-Analysis of Data on the Arg399Gln Polymorphism

The meta-analysis of a possible association between the XRCC1 gene polymorphisms and pancreatic cancer risk was summarized in Table 2. For XRCC1 gene Arg399Gln polymorphism, 9 case-control studies including 2067 cases and 4426 controls were included in the meta-analysis. Based on the total populations, none of the five genetic models indicated a significant association (allelic model, OR 1.111, 95% CI 0.954–1.293, *P* = 0.177; heterozygous model, OR 1.083, 95% CI 0.961–1.220, *P* = 0.190; homozygous model, OR 1.212, 95% CI 0.906–1.620, *P* = 0.195; dominant model, OR 1.123, 95% CI 0.939–1.342, *P* = 0.204; recessive model, OR 1.135, 95% CI 0.950–1.356, *P* = 0.162). We performed a stratified analysis based on ethnicity, and no significant association was observed between the Arg399Gln polymorphism and pancreatic cancer risk in Asians or Caucasians.

**Table 2:** Main results on association between XRCC1 gene polymorphisms and pancreatic cancer risk

Comparisons	No. of Studies	Test of association			Analysis model	Test of heterogeneity		
		OR	95% CI	*P-value*		χ^2^	*P-value*	I^2^ (%)
XRCC1 gene Arg399Gln polymorphism in total populations								
Allelic (A versus G)	9	1.111	0.954–1.293	0.177	Random	22.70	0.004	64.8%
Heterozygous (GA versus GG)	9	1.083	0.961–1.220	0.190	Fixed	12.33	0.137	35.1%
Homozygous (AA versus GG)	9	1.212	0.906–1.620	0.195	Random	16.18	0.040	50.5%
Dominant (AA + GA versus GG)	9	1.123	0.939–1.342	0.204	Random	18.16	0.020	55.9%
Recessive (AA versus GA + GG)	9	1.135	0.950–1.356	0.162	Fixed	11.44	0.178	30.1%
XRCC1 gene Arg399Gln polymorphism in HWE								
Allelic (A versus G)	8	1.065	0.921–1.230	0.396	Random	16.38	0.022	57.3%
Heterozygous (GA versus GG)	8	1.063	0.939–1.203	0.335	Fixed	11.13	0.133	37.1%
Homozygous (AA versus GG)	8	1.089	0.893–1.327	0.400	Fixed	10.96	0.140	36.1%
Dominant (AA + GA versus GG)	8	1.080	0.902–1.292	0.405	Random	14.79	0.039	52.7%
Recessive (AA versus GA + GG)	8	1.070	0.887–1.290	0.481	Fixed	7.23	0.405	3.2%
XRCC1 gene Arg399Gln polymorphism in Asian populations								
Allelic (A versus G)	6	1.155	0.900–1.483	0.256	Random	16.81	0.005	70.2%
Heterozygous (GA versus GG)	6	1.166	0.985–1.382	0.075	Fixed	7.11	0.213	29.7%
Homozygous (AA versus GG)	6	1.303	0.784–2.165	0.307	Random	12.42	0.029	59.7%
Dominant (AA + GA versus GG)	6	1.180	0.906–1.537	0.219	Random	12.01	0.035	58.4%
Recessive (AA versus GA + GG)	6	1.269	0.950–1.695	0.106	Fixed	9.61	0.087	48.0%
XRCC1 gene Arg399Gln polymorphism in Caucasians populations								
Allelic (A versus G)	3	1.027	0.917–1.151	0.643	Fixed	3.47	0.177	42.3%
Heterozygous (GA versus GG)	3	1.007	0.852–1.191	0.932	Fixed	3.74	0.154	46.5%
Homozygous (AA versus GG)	3	1.066	0.836–1.358	0.607	Fixed	2.09	0.352	4.3%
Dominant (AA + GA versus GG)	3	1.038	0.823–1.309	0.753	Random	4.24	0.120	52.9%
Recessive (AA versus GA + GG)	3	1.063	0.849–1.331	0.596	Fixed	0.79	0.673	0.0%
XRCC1 gene Arg194Trp polymorphism in total populations								
Allelic (T versus C)	7	1.223	0.939–1.592	0.136	Random	28.88	0.000	79.2%
Heterozygous (CT versus CC)	7	1.229	0.881–1.713	0.225	Random	29.30	0.000	79.5%
Homozygous (TT versus CC)	7	1.140	0.832–1.563	0.414	Fixed	4.83	0.565	0.0%
Dominant (TT + CT versus CC)	7	1.247	0.900–1.726	0.184	Random	30.46	0.000	80.3%
Recessive (TT versus CT + CC)	7	1.130	0.831–1.534	0.436	Fixed	3.33	0.767	0.0%
XRCC1 gene Arg194Trp polymorphism in HWE								
Allelic (T versus C)	5	1.315	0.927–1.865	0.125	Random	23.28	0.000	82.8%
Heterozygous (CT versus CC)	5	1.390	0.919–2.103	0.118	Random	21.52	0.000	81.4%
Homozygous (TT versus CC)	5	1.113	0.758–1.635	0.583	Fixed	3.46	0.483	0.0%
Dominant (TT + CT versus CC)	5	1.387	0.918–2.098	0.121	Random	22.99	0.000	82.6%
Recessive (TT versus CT + CC)	5	1.065	0.733–1.546	0.743	Fixed	2.21	0.698	0.0%
XRCC1 gene Arg194Trp polymorphism in Asian populations								
Allelic (T versus C)	5	1.080	0.875–1.332	0.475	Random	9.72	0.045	58.8%
Heterozygous (CT versus CC)	5	1.048	0.809–1.357	0.723	Random	8.55	0.073	53.2%
Homozygous (TT versus CC)	5	1.105	0.798–1.532	0.547	Fixed	4.05	0.400	1.2%
Dominant (TT + CT versus CC)	5	1.075	0.824–1.401	0.595	Random	9.92	0.042	59.7%
Recessive (TT versus CT + CC)	5	1.104	0.805–1.516	0.539	Fixed	2.92	0.572	0.0%
XRCC1 gene Arg194Trp polymorphism in Caucasians populations								
Allelic (T versus C)	2	1.707	0.646–4.512	0.281	Random	12.84	0.000	92.2%
Heterozygous (CT versus CC)	2	1.840	0.587–5.768	0.295	Random	14.56	0.000	93.1%
Homozygous (TT versus CC)	2	1.815	0.521–6.321	0.349	Fixed	0.20	0.656	0.0%
Dominant (TT + CT versus CC)	2	1.825	0.600–5.546	0.289	Random	14.46	0.000	93.1%
Recessive (TT versus CT + CC)	2	1.607	0.463–5.584	0.455	Random	0.08	0.775	0.0%
XRCC1 gene Arg280His polymorphism in total populations								
Allelic (A versus G)	3	0.956	0.589–1.550	0.854	Random	8.40	0.015	76.2%
Heterozygous (GA versus GG)	3	0.772	0.592–1.008	0.057	Fixed	2.97	0.227	32.6%
Homozygous (AA versus GG)	3	1.216	0.630–2.349	0.560	Fixed	3.60	0.165	44.5%
Dominant (AA + GA versus GG)	3	0.885	0.565–1.385	0.592	Random	5.86	0.053	65.9%
Recessive (AA versus GA + GG)	3	1.261	0.653–2.432	0.490	Fixed	3.07	0.215	34.9%
XRCC1 gene Arg280His polymorphism in HWE								
Allelic (A versus G)	2	0.743	0.576–0.958	0.022	Fixed	0.04	0.833	0.0%
Heterozygous (GA versus GG)	2	0.701	0.525–0.936	0.016	Fixed	0.00	0.996	0.0%
Homozygous (AA versus GG)	2	0.794	0.339–1.859	0.595	Fixed	0.98	0.322	0.0%
Dominant (AA + GA versus GG)	2	0.710	0.537–0.939	0.016	Fixed	0.03	0.873	0.0%
Recessive (AA versus GA + GG)	2	0.856	0.367–1.999	0.720	Fixed	0.90	0.342	0.0%
XRCC1 gene c.1517G > C polymorphism in total populations								
Allelic (C versus G)	2	1.252	1.064–1.473	0.007	Fixed	0.52	0.471	0.0%
Heterozygous (GC versus GG)	2	1.025	0.786–1.335	0.858	Fixed	0.01	0.903	0.0%
Homozygous (CC versus GG)	2	1.800	0.976–3.322	0.060	Random	2.41	0.120	58.6%
Dominant (CC + GC versus GG)	2	1.187	0.924–1.526	0.180	Fixed	0.00	0.973	0.0%
Recessive (CC versus GC + GG)	2	1.677	0.876–3.211	0.119	Random	4.63	0.031	78.4%
XRCC1 gene c.1471G > A polymorphism in total populations								
Allelic (A versus G)	2	0.914	0.467–1.789	0.794	Random	15.61	0.000	93.6%
Heterozygous (GA versus GG)	2	0.948	0.547–1.641	0.848	Random	5.51	0.019	81.9%
Homozygous (AA versus GG)	2	0.860	0.210–3.513	0.833	Random	12.31	0.000	91.9%
Dominant (AA + GA versus GG)	2	0.915	0.444–1.886	0.810	Random	10.66	0.001	90.6%
Recessive (AA versus GA + GG)	2	0.879	0.277–2.789	0.827	Random	8.92	0.003	88.8%

### Meta-Analysis of Data on the Arg194Trp Polymorphism

For XRCC1 gene Arg194Trp polymorphism, 7 case-control studies including 1594 cases and 3517 controls were included in the meta-analysis. There was no significant association between XRCC1 gene Arg194Trp polymorphism and pancreatic cancer risk (allelic model, OR 1.223, 95% CI 0.939–1.592, *P* = 0.136; heterozygous model, OR 1.229, 95% CI 0.881–1.713, *P* = 0.225; homozygous model, OR 1.140, 95% CI 0.832–1.563, *P* = 0.414; dominant model, OR 1.247, 95% CI 0.900–1.726, *P* = 0.184; recessive model, OR 1.130, 95% CI 0.831–1.534, *P* = 0.436). We performed a stratified analysis based on ethnicity, and no significant association was observed between the Arg194Trp polymorphism and pancreatic cancer risk in Asians or Caucasians.

### Meta-Analysis of Data on the Arg280His Polymorphism

For XRCC1 gene Arg280His polymorphism, 3 case-control studies including 918 cases and 1147 controls were included in the meta-analysis. Based on the total populations, there was no significant association between XRCC1 gene Arg280His polymorphism and pancreatic cancer risk (allelic model, OR 0.956, 95% CI 0.589–1.550, *P* = 0.854; heterozygous model, OR 0.772, 95% CI 0.592–1.008, *P* = 0.057; homozygous model, OR 1.216, 95% CI 0.630–2.349, *P* = 0.560; dominant model, OR 0.885, 95% CI 0.565–1.385, *P* = 0.592; recessive model, OR 1.261, 95% CI 0.653–2.432, *P* = 0.490). However, based on the HWE, there was significant association between XRCC1 gene Arg280His polymorphism and pancreatic cancer risk (allelic model, OR 0.743, 95% CI 0.576–0.958, *P* = 0.022; heterozygous model, OR 0.701, 95% CI 0.525–0.936, *P* = 0.016; dominant model, OR 0.710, 95% CI 0.537–0.939, *P* = 0.016). Stratified analysis based on ethnicity was not performed due to limited studies.

### Meta-Analysis of Data on the C.1517G > C Polymorphism

For XRCC1 gene c.1517G > C polymorphism, 2 case-control studies including 688 cases and 690 controls were included in the meta-analysis. There was significant association between XRCC1 gene c.1517G > C polymorphism and pancreatic cancer risk (allelic model, OR 1.252, 95% CI 1.064–1.473, *P* = 0.007). But no significant association was observed between the c.1517G > C polymorphism and pancreatic cancer risk under other four genetic models.

### Meta-Analysis of Data on the c.1471G > A Polymorphism

For XRCC1 gene c.1471G > A polymorphism, 2 case-control studies including 626 cases and 648 controls were included in the meta-analysis. There was no significant association between XRCC1 gene c.1471G > A polymorphism and pancreatic cancer risk (allelic model, OR 0.914, 95% CI 0.467–1.789, *P* = 0.794; heterozygous model, OR 0.948, 95% CI 0.547–1.641, *P* = 0.848; homozygous model, OR 0.860, 95% CI 0.210–3.513, *P* = 0.833; dominant model, OR 0.915, 95% CI 0.444–1.886, *P* = 0.810; recessive model, OR 0.879, 95% CI 0.277–2.789, *P* = 0.827).

### Sensitivity Analysis

In order to assess the robustness of the meta-analysis results, we carried out a sensitivity analysis. Our results showed that pooled ORs were not materially altered, which suggested that no individual study significantly affected the pooled results.

### Publication Bias

Begg’s funnel plots and Egger’s regression method were used to assess publication bias statistically. For the Arg399Gln polymorphism, the results of Egger’s linear regression test did not provide statistical evidence of publication bias (*P* > 0.05) (Table 3). Funnel plot analyses were also not generally indicative of any strong publication bias because visual inspection of funnel plots did not show asymmetry for all comparison models, indicating the strength of the results.

**Table 3:** Results of publication bias by Egger’s linear regression test for the Arg399Gln polymorphism

Egger’s test	A versus G	GA versus GG	AA versus GG	AA + GA versus GG	AA versus GA + GG
t	0.34	0.06	0.25	0.19	0.23
*P*-value	0.746	0.955	0.812	0.854	0.824

## Discussion

Emerging evidences have shown that common genetic polymorphisms in XRCC1 gene may be associated with the development of pancreatic cancer, but individually published studies and previous meta-analyses revealed inconclusive results. In the present study, to obtain a more precise estimation of the association between the XRCC1 gene polymorphisms and pancreatic cancer risk, we conducted a systematic review with meta-analysis by critically reviewing all published studies.

For XRCC1 gene Arg399Gln polymorphism, we found no strong evidence of association with susceptibility to pancreatic cancer. Wang et al. [17] study was considered as deviation from HWE. We performed a stratified analysis based on HWE, and the pooled data also demonstrated that there was no evidence of association between the Arg399Gln polymorphism and the susceptibility of pancreatic cancer. We performed a subgroup analysis based on ethnicity, and the pooled data also demonstrated that there was no evidence of association between the Arg399Gln polymorphism and the susceptibility of pancreatic cancer in Asians or Caucasians. However, it was not in agreement with the result of Jiang et al. [18] study in which the pooled data demonstrated that XRCC1 gene Arg399Gln (rs25487) polymorphism is associated with pancreatic cancer risk in Asians. Sensitivity analysis demonstrated that pooled ORs were not materially altered, indicating that our results were robust and reliable. After evaluating the publication bias by Begg’s funnel plots and Egger’s regression method, we did not detect a publication bias based on total population, indicating the strength of the results.

In this meta-analysis, a total of 7 case-control studies were analyzed to provide a comprehensive assessment of the association between Arg194Trp polymorphism and pancreatic cancer risk. Our results did not support a genetic association between Arg194Trp and susceptibility to pancreatic cancer. Neither allele frequency nor genotype distribution was significantly associated with susceptibility to pancreatic cancer. Since the incidence of gene polymorphisms may vary between different ethnic groups and this variation may interfere with the detection of minor effect of SNPs on pancreatic cancer risk, subgroup analysis according to ethnicity was performed to further explore the potential association between Arg194Trp and the risk of pancreatic cancer. However, even within the same ethnic group, no association of statistical significance was observed.

For XRCC1 gene Arg280His polymorphism, 3 case-control studies including 918 cases and 1147 controls were included in our systematic review. Wang et al. [17] study was considered as deviation from HWE. Results from our stratified analysis based on HWE showed that there was a robust significant association between Arg280His polymorphism and pancreatic cancer risk. However, previous meta-analysis conducted by Shen et al. [19] reported there was no evidence that XRCC1 gene Arg280His polymorphism are associated with pancreatic cancer risk. Their meta-analysis with only one eligible study [20] has insufficient statistical power to detect a small effect, while our meta-analysis included 3 case-control studies including 918 cases and 1147 controls. So the reasons for inconsistent results might be that larger sample sizes may lead to the identification of statistically significant correlation.

Two case-control studies on the relationship between c.1517G > C polymorphism and susceptibility to pancreatic cancer were included in our systematic review. Zhao et al. [21] and Hou et al. [22] reported that the XRCC1 c.1517G > C variant is significantly associated with pancreatic cancer susceptibility in the Chinese population. According to the current evidence-based literature, we thought that there was significant association between XRCC1 gene c.1517G > C polymorphism and pancreatic cancer risk.

Similarly, Two case-control studies on the relationship between c.1471G > A polymorphism and susceptibility to pancreatic cancer were included in our meta-analysis. Retracted article conducted by Chen et al. [23] reported that the AA genotype was associated with the decreased risk of developing pancreatic cancer compared to GG wild genotype. Hou et al. [22] reported that the variant A allele frequency was borderline higher among patients in the pancreatic cancer group compared with the control group. To our surprise, we failed to find any association between c.1471G > A polymorphism and the risk for pancreatic cancer in any of the examined genetic models. For the limited studies, the results should be treated with caution.

However, several limitations of this meta-analysis should be considered. Firstly, our exclusion of papers published in languages other than English and Chinese might have biased our results. Secondly, the sample size is not large enough, especially for subgroup analysis. Thus, the observed significant associations in some subgroup analysis may be not accurate. Lastly, lack of available data prevented us from performing additional subgroup analyses by age, gender, alcohol consumption and other risk factors, which could be potential factors influencing the evaluation of the associations between SNPs in XRCC1 gene and pancreatic cancer risk.

In conclusion, the present meta-analysis suggested that Arg280His and c.1517G > C polymorphisms in XRCC1 gene were associated with pancreatic cancer risk. Considering the limited sample size and ethnicity enrolled in this meta-analysis, further larger scaled studies should be performed to validate the association.

## Electronic supplementary material

ESM 1 (DOCX 13 kb)
