# Supplementary material for: Association between Polymorphisms of X-Ray Repair Cross Complementing Group 1 Gene and Pancreatic Cancer Risk: a Systematic Review with Meta-Analysis
Source: Pathol Oncol Res. 2017 Dec 28;25(3):897–904. doi: 10.1007/s12253-017-0364-6 (PMC6614153; doi:10.1007/s12253-017-0364-6)
Supplement: Supplementary file 1 — (DOCX 13 kb) [file 12253_2017_364_MOESM1_ESM.docx]

| **S1 Table. The detailed search strategies and results.** | | | |
| --- | --- | --- | --- |
| **Database** | **Last Update** | **Search Strategy** | **results** |
| PubMed | February 12, 2017 | ((((((((XRCC1[All Fields] OR rs25487[All Fields]) OR Arg399Gln[All Fields]) OR rs1799782[All Fields]) OR Arg194Trp[All Fields]) OR rs25489[All Fields]) OR Arg280His[All Fields]) OR c.1517G>C[All Fields]) OR c.1471G>A[All Fields]) AND (("pancreatic neoplasms"[MeSH Terms] OR ("pancreatic"[All Fields] AND "neoplasms"[All Fields]) OR "pancreatic neoplasms"[All Fields] OR ("pancreatic"[All Fields] AND "cancer"[All Fields]) OR "pancreatic cancer"[All Fields]) OR ("Pancreatic Carcinoma"[Supplementary Concept] OR "Pancreatic Carcinoma"[All Fields] OR "pancreatic carcinoma"[All Fields])) | 22 |
| Embase | February 12, 2017 | #1 'xrcc1' OR 'rs25487' OR 'arg399gln' OR 'rs1799782' OR 'arg194trp' OR 'rs25489' OR 'arg280his'  #2 'pancreatic cancer' OR 'pancreatic carcinoma'  #3 #1 AND #2 | 27 |
| The Cochrane Library | February 12, 2017 | #1 'xrcc1' OR 'rs25487' OR 'arg399gln' OR 'rs1799782' OR 'arg194trp' OR 'rs25489' OR 'arg280his'  #2 'pancreatic cancer' OR 'pancreatic carcinoma'  #3 #1 AND #2 | 1 |
| Web of Science | February 12, 2017 | TOPIC: ('xrcc1' OR 'rs25487' OR 'arg399gln' OR 'rs1799782' OR 'arg194trp' OR 'rs25489' OR 'arg280his') AND TOPIC: ('pancreatic cancer' OR 'pancreatic carcinoma') | 8 |
